# Retroperitoneal Cyst: An Uncommon Presentation of Filariasis

**DOI:** 10.1155/2015/674252

**Published:** 2015-11-19

**Authors:** Senthil Ganesan, Saurabh Galodha, Rajan Saxena

**Affiliations:** Department of Surgical Gastroenterology, Sanjay Gandhi Post Graduate Institute of Medical Sciences, Lucknow 226014, India

## Abstract

Primary retroperitoneal parasitic cysts are rare. Here we report about a middle aged male patient from rural north India with a recent onset of central abdominal retroperitoneal lump, pain, and fever. After surgical resection due to diagnostic uncertainty, at histopathology, it turned out be a filarial cyst. After receiving a course of diethylcarbamazine, the patient is asymptomatic at 4 months' follow-up.

## 1. Introduction

Primary retroperitoneal parasitic cysts are uncommon [1, 2]. Mostly these are echinococcal cysts [3]. Retroperitoneal filarial cysts are very rare even in endemic areas like India and there are only few case reports [4, 5]. Here we report an unusual presentation of filariasis as retroperitoneal cyst.

## 2. Case Presentation

A 35-year-old gentleman, from rural north India, presented with a progressive, central abdominal lump for one-month duration. Three weeks after the appearance of the lump, he developed intermittent high grade fever and dull aching abdominal pain. He did not have any significant past illness or comorbidities. On examination, he had a moderately tender abdominal lump predominantly in the umbilical region. Apart from nontender thickening of the right spermatic cord, there was no scrotal or testicular abnormality. The inguinal nodes were barely palpable and there was no lower limb edema.

**Figure 1:**
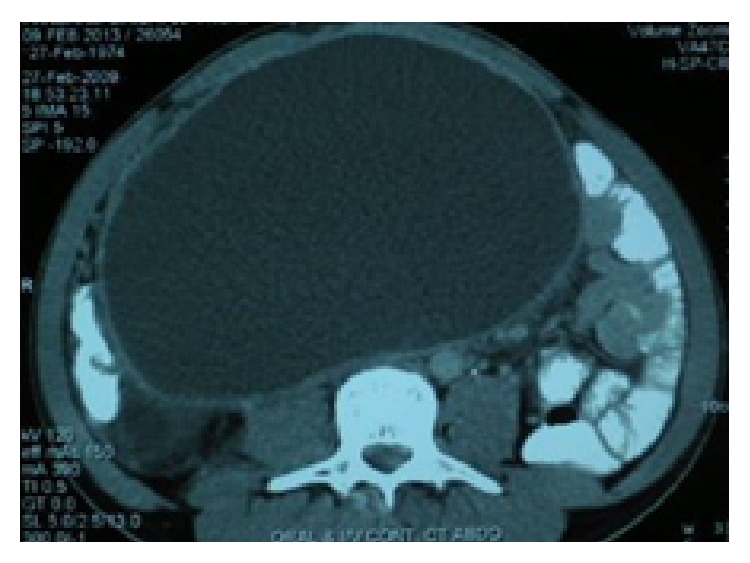
Contrast enhanced CT scan showing retroperitoneal cyst.

Complete blood counts and blood chemistry were within normal limits. Blood culture was sterile. Abdominal sonography revealed a large, well defined, unilocular cyst of 20 × 14 × 23 cm size, located behind the right colon and terminal ileum. There were no features suggestive of an abscess. The rest of the organs were normal. Contrast enhanced CT scan confirmed the sonographic findings without adding any other information. The origin and etiology of the cyst could not be delineated (Figure 1).

**Figure 2:**
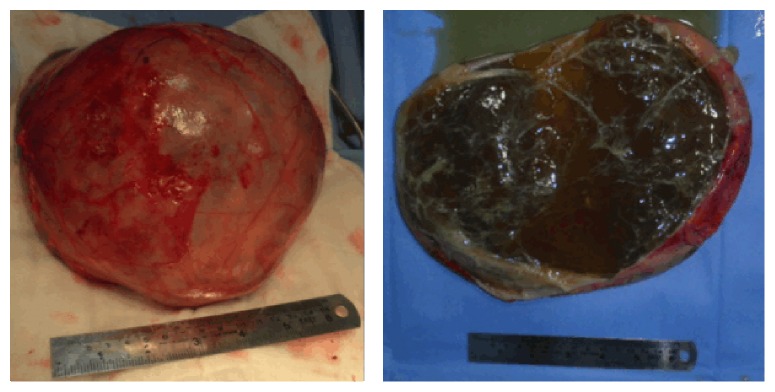
Excised retroperitoneal filarial cyst and its cut section.

Due to diagnostic uncertainty and association of symptoms like pain, the abdomen was explored revealing a large retroperitoneal cyst behind the right colon, mesocolon, and terminal ileum, stretching the organs over it. As there was well defined avascular plane around the cyst, the cyst could be excised completely without any spillage. A conglomeration of grossly dilated lymphatics was seen close to upper pole of the cyst and excised along with the cyst. The rest of the organs were normal. The cut section of the cyst revealed a thick walled cyst containing minute filamentous septations and mucoid material (Figure 2). Postoperative recovery was uneventful.

**Figure 3:**
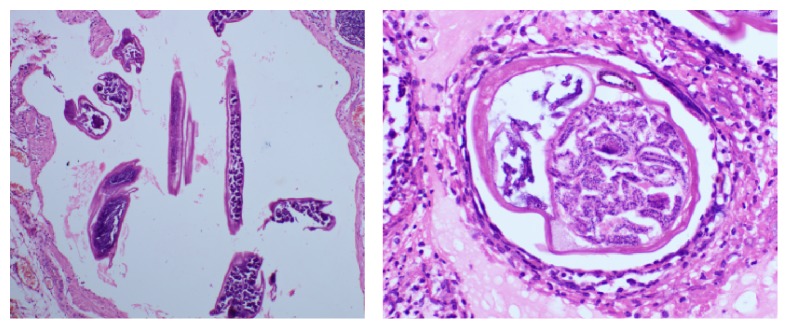
Histopathology of cyst showing microfilaria.

Cyst fluid analysis showed total cell count of 95/cmm with 60% lymphocytes and 40% neutrophils. Cyst fluid culture was sterile. Histopathology of the cyst showed numerous cystic spaces packed with microfilariae and dense mixed inflammatory infiltrate in the surrounding tissues (Figure 3). It was a retroperitoneal filarial cyst! After a course of diethylcarbamazine (100 mg t.i.d. for 3 weeks), he is asymptomatic at 4 months' follow-up.

## 3. Discussion

Primary retroperitoneal cysts are rare, often asymptomatic, and incidentally detected. They have been classified as (a) urogenital cysts, (b) mesocolic cysts, (c) cysts arising in cell inclusions, (d) traumatic cysts, (e) parasitic cysts, and (f) lymphatic cysts, depending on the origin and histology [1, 2]. Often primary retroperitoneal parasitic cysts are echinococcal cysts and Aydinli et al. reported a series of 14 cases [3].

Filariasis presenting as a retroperitoneal cyst is extremely uncommon. Reported incidence of filarial retroperitoneal cyst in hospitalized patients is 1/105000 [6, 7]. To our knowledge, there are only four case reports of filariasis presenting as retroperitoneal cyst, abscess, or mass and most of them are from India [4, 5, 8, 9]. There are also reports of filariasis presenting as abscess [8] and heterogenous mass mimicking retroperitoneal tumor [9].

Kapoor et al. reported about a patient with similar presentation except the duration of symptoms was longer (6 months) and patient also had bilateral hydrocele [4]. They established the diagnosis by fine needle aspiration cytology (FNAC). As it did not regress after 4 weeks of diethylcarbamazine therapy, they ultimately needed to excise the cyst. Giri et al. reported about a young male patient presenting with heterogenous mass at left iliac fossa along with left sided hydrocele and hydronephrosis of left kidney [9]. After confirming the diagnosis by FNAC, the patient was treated with antifilarial therapy which led to almost complete resolution of the cyst.

Among all 8 species of filarial worms, the most common organism encountered in India is* Wuchereria bancrofti*. Common presentations are asymptomatic microfilaremia, acute adenolymphangitis, hydrocele, and lymphedema [10]. Recent onset of retroperitoneal cyst, without other common manifestations of filariasis like hydrocele and lymphedema, is the peculiar presentation of our patient. We decided to perform surgery straight away without FNAC, as the patient had large symptomatic cyst with diagnostic uncertainty. If we analyze retrospectively, high grade fever with normal leucocyte count, no bacteremia on blood culture, and a subtle sign of right sided spermatic cord thickening in a patient from the background of rural north India (endemic areas) could have been the clues. Imaging rarely helps to clinch the diagnosis. Often the diagnosis is made either by FNAC or histopathology after excision like in our case. In smaller cysts, if confirmed by preoperative investigations like FNAC, one can start with medical treatment first and surgical excision may be reserved for persistent symptomatic cysts.

## 4. Conclusion

Even though filarial retroperitoneal cysts are uncommon, in endemic areas it must be included in the differential diagnosis of the retroperitoneal cysts.
